# Indigenous fungi inoculation drives organic carbon accumulation and phosphorus transformation in coal gangue-based artificial soil: role of mineral-bioactivation

**DOI:** 10.3389/fmicb.2026.1758978

**Published:** 2026-02-04

**Authors:** Huofeng Zhang, Runan Xu, Yijie Quan, Donghe Xue, Ling Hu, Yan Yang, Ying Dong, Wei Wang, Huijuan Bo, Qiang Zhang, Minggang Xu, Dongsheng Jin

**Affiliations:** 1 College of Resources and Environment, Shanxi Agricultural University, Taiyuan, China; 2 Soil Health Laboratory in Shanxi Province, Taiyuan, China

**Keywords:** ecological restoration, microbial activation, mining-derived substrates, nutrient mobilization, organic acids

## Abstract

Coal gangue accumulation occupies extensive land areas, causing severe environmental pollution and degrading soil quality in mine reclamation sites. In this study, we aimed to identify the optimal inoculum concentration that maximizes physicochemical improvement and biological stability. The bioactivation effects of indigenous fungal inocula (1–5%) on coal gangue (C) and coal gangue-loess mixtures (M) were assessed by analyzing fungi abundance, pH, electrical conductivity (EC), total organic carbon, available phosphorus, and mineral composition. The indigenous fungi J-Z, isolated from coal gangue dumps, exhibited optimal bioactivation performance at moderate inoculum concentrations (3–4%). The pH initially decreased during days 7–14 and subsequently stabilized, with the 3% inoculum (M3) and 4% (M4) inoculum treatments exhibiting the strongest buffering capacity. EC increased with inoculum concentration until reaching equilibrium. Higher inoculum concentrations enhanced total organic carbon accumulation in both the C and M systems, especially under M3 and M4 treatments. Available phosphorus increased with inoculum concentration, peaking in M4 (3.0–3.5 mg·kg^−1^) after 60 days. XRD analysis revealed a significant transformation from Strengite to Monetite in the M4 treatment. Organic acid analysis indicated that citric acid was the primary driving force behind this transformation. Overall, M4 achieved the optimal balance between fungi activation and physicochemical stability. This study establishes a scientific framework for the sustainable utilization of coal gangue as a reclamation substrate through coupled biochemical processes involving fungi activation, mineral transformation, and nutrient stabilization.

## 1 Introduction

Coal gangue represents the principal solid waste generated during coal mining and washing (Yang et al., 2024). Its large-scale disposal leads to environmental degradation, spontaneous combustion, and reduced land availability (Yutao, 2022). Furthermore, mine development reduces the availability of high-quality topsoil, which greatly hinders ecological restoration and land reuse (Ribeiro et al., 2016). Although coal gangue has been employed in soil improvement and land reclamation, its physicochemical properties strongly determine its utilization rate and remediation efficiency. Generally, coal gangue has high inert mineral contents, such as Al₂O₃ and SiO₂; extremely low available nutrient contents, such as organic matter and available nitrogen, phosphorus, and potassium; and a weak nutrient-supplying ability (Liu and Wang, 2025). Additionally, coal gangue is often neutral to weakly acidic in reaction. Sulfide minerals, such as pyrite, may form acidic products when weathered or oxidized, exacerbating soil acidification (Rambabu et al., 2020; Li, 2021). Therefore, enhancing the environmental compatibility of coal gangue and its functionality as a soil amendment has become an important research topic in soil remediation (Tang et al., 2024; Chengyong et al., 2025).

Bioactivation technology utilizing coal gangue has been applied extensively in environmental remediation (Wang et al., 2022; Guan et al., 2024; Xu et al., 2025). However, few studies have reported the indigenous microbial inocula. Indigenous microorganisms can facilitate the dissolution and transformation of minerals through community-wide activities, enhancing the physicochemical properties and biological activities of coal gangue (Dauptain et al., 2020; Li et al., 2024). Adding microbial inoculants can enhance the fertility of coal gangue and microbial community diversity of the treated soil, thereby improving the remediation effect (Akimbekov et al., 2022). However, few systematic studies have characterized the effects of indigenous inoculum dosage on coal gangue bioactivity and remediation (Tao et al., 2024), as most prior work has emphasized strain screening and single-function analysis (He et al., 2017; Ruan et al., 2023; Bi et al., 2025). Different inoculum concentrations may have different effects on mineral transformation and microbial activity, which may further influence remediation (Fomina and Skorochod, 2020). Nevertheless, few studies have considered the optimization of inoculum dosage (Kane et al., 2020).

Coal gangue is faced with multiple limitations of nutrient deficiency and strong mineral stability, which seriously restrict the early biological colonization and soil formation process. Although a variety of microorganisms are involved in the reclamation process, fungi play a particularly important role in the mining disturbance matrix due to their strong tolerance to oligotrophic and metal stress environments. Filamentous fungi can penetrate dense coal gangue matrix through mycelial growth, and promote mineral weathering, carbon conversion, and aggregate formation by secreting organic acids, which often occupy the advantage of microbial biomass in the early stage of reclamation (Wang et al., 2021; Ji et al., 2022). The synergistic effects of fungi, bacteria, archaea, and protists may be different from the effects of single fungi. Focusing on fungi helps to more clearly analyze the key mechanisms in the initial biogeochemical transformation of coal gangue matrix (Borkar et al., 2023; Wang et al., 2024a). Therefore, this study focuses on fungal inoculation, aiming to clarify its specific role in regulating the physical and chemical properties of coal gangue and the coal gangue-soil mixed system.

In this study, simulated experiments using coal gangue sampled from the Guxian–Tunlan mining reclamation area were conducted to systematically determine the bioactivity, mineral transformation, and physicochemical properties of coal gangue (C system) and mixtures with loess (M system) containing different concentrations of J-Z indigenous fungi inoculums. We aimed to determine the optimal inoculum concentration with the greatest physicochemical improvement and biological stability, providing a theoretical basis and technical support for the utilization of coal gangue and ecological reclamation of mining areas.

## 2 Materials and methods

### 2.1 Experimental materials

The coal gangue was collected from the experimental area located in the Tunlan mining reclamation area of Gujiao, Taiyuan City, Shanxi Province (Longitude 112°06′E, Latitude 37°53′N). The area has an annual average temperature of 9.5 °C and an average annual precipitation of 460 mm, characterized by a temperate continental monsoon climate. The area is a coal reclamation area affected by underground mining activities. After the mining operation, the coal gangue is excavated as backfill material, and the loess is covered in the reclamation process. The loess was collected from the surrounding mountainous villages.

Enrichment medium: PDA medium (Potato Dextrose Agar): 200 g of potatoes, 20 g of glucose, 18 g of agar, 1,000 mL of purified water (Syamsia et al., 2021), used for cultivating J-Z fungus.

**Figure 1:**
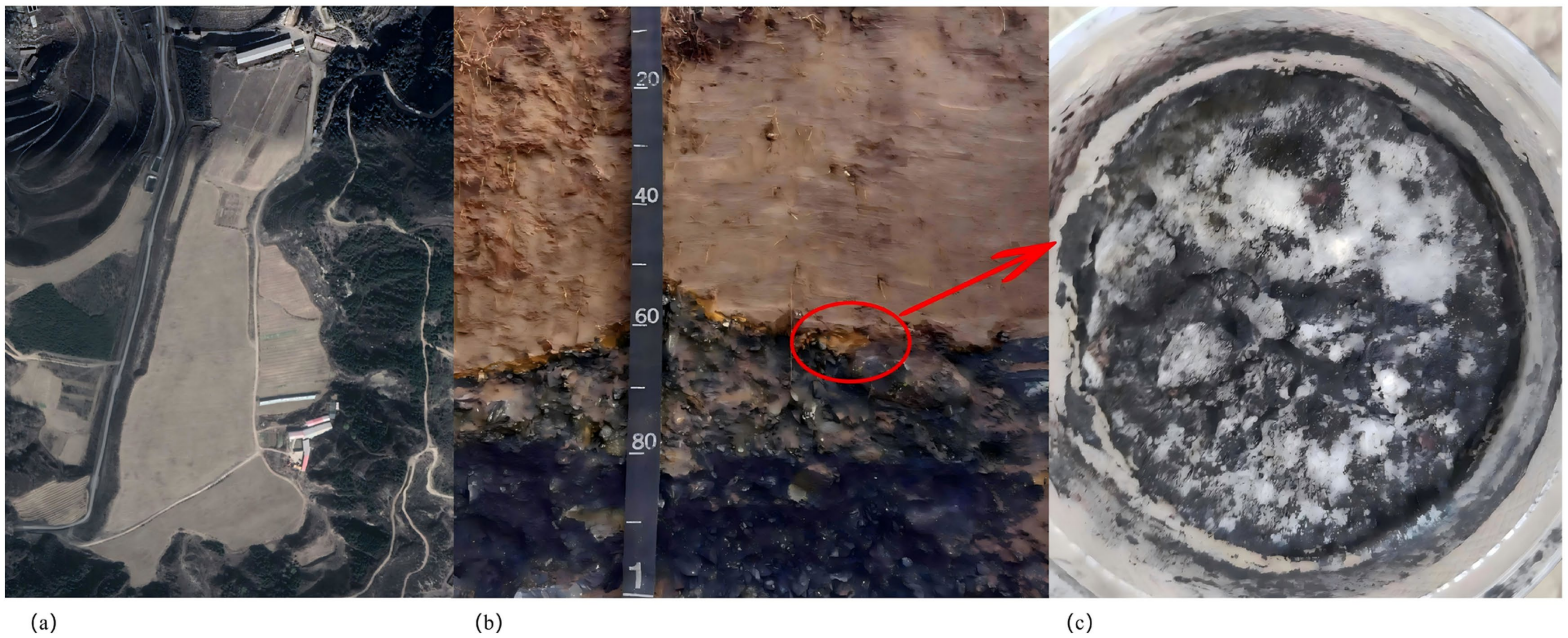
Gujiao Tunlan mining area reclamation area **(a)**, shows the interface between coal gangue and soil in the reclaimed mining area **(b)**, and shows the strains selected after screening **(c)**.

The indigenous fungi strain of J-Z fungi liquid was obtained by sampling, screening, isolation, and purification from the reclamation profile of the Gujiao coal gangue mining area (Figure 1).

### 2.2 Experimental methods

Material preparation: Coal gangue was ground using a grinder and sieved through a 100-mesh screen to obtain coal gangue powder. Loess was also sieved through a 100-mesh screen and dried in an oven at 105 °C (Schroeder et al., 2021; Even et al., 2025). Subsequently, 100 g of coal gangue and a mixture of coal gangue and loess (at a mass ratio of 80:20 g) were each placed into 150 mL Erlenmeyer flasks (Li et al., 2023) and sterilized by autoclaving at 121 °C (King et al., 2024).

Experimental design: Indigenous fungi inoculant (strain J-Z) was added to each 150 mL Erlenmeyer flask containing 100 g of either pure coal gangue or the coal gangue-loess mixture at dosages of 1 mL, 2 mL, 3 mL, 4 mL, and 5 mL, respectively. Each treatment was prepared in five replicates. The inoculum concentration was 1.6 × 10^9^ CFU·mL^−1^. All samples were incubated in a constant temperature chamber, maintained at 28 °C (El-Nagar et al., 2024; Amos et al., 2025; Zhu et al., 2025), and observations were made at different time points of 3, 7, 15, 30, and 60 d.

#### 2.2.1 Experimental design

The C treatment consisted of pure coal gangue, while the M treatment represented the mixture of coal gangue and loess (80:20 g) Table 1. Treatments C0–C5 and M0–M5 corresponded to the addition of J-Z fungi inoculant at concentrations of 0–5%, respectively (Rashid et al., 2011; Korayem et al., 2025; Ruslan et al., 2025). Each treatment was conducted in five replicates, and the moisture content was maintained at 18 g.

**Table 1:** Experimental treatments.

Serial number	Coal gangue powder: raw loess		J-Z	Sterile water
C0	100 g	0	0	18 mL
C1	100 g	0	1 mL	17 mL
C2	100 g	0	2 mL	16 mL
C3	100 g	0	3 mL	15 mL
C4	100 g	0	4 mL	14 mL
C5	100 g	0	5 mL	13 mL
M0	80 g	20 g	0	18 mL
M1	80 g	20 g	1 mL	17 mL
M2	80 g	20 g	2 mL	16 mL
M3	80 g	20 g	3 mL	15 mL
M4	80 g	20 g	4 mL	14 mL
M5	80 g	20 g	5 mL	13 mL

#### 2.2.2 Measurement indicators

The number of J-Z fungi was determined using the plate count method to assess the impact of inoculant addition on J-Z fungi activity in the substrate (Weitzel et al., 2021). pH and EC were measured using soil and deionized water at ratios of 1:2.5 and 1:5 (w/v), respectively. The pH value was determined using a pH meter, and the EC value was measured with a conductivity meter to characterize changes in soluble salts and ion strength. The potassium dichromate oxidation method with external heating is used to determine total organic carbon (TOC) content (Coal gangue is a mining-derived solid byproduct rather than a natural soil material. Therefore, its organic carbon, mainly composed of residual coal, kerogen-like carbon, and highly condensed aromatic carbon, which is reported as total organic carbon (TOC)). The AP content was extracted using a 0.5 mol·L^−1^ NaHCO₃ extraction method, and then determined by the molybdenum-antimony colorimetric method. The mineral composition of the sample was analyzed using X-ray diffraction (XRD) technology, focusing on phosphate minerals such as Brushite, Monetite, Strengite, and Variscite. Their relative contents were calculated based on the ratio of characteristic peak areas (Chen et al., 2021). This study used high-performance liquid chromatography (HPLC) to determine the organic acids (such as citric acid, malic acid, fumaric acid, and glutamic acid) metabolized by natural fungi in the system, in order to elucidate their role in mineral dissolution and nutrient release.

#### 2.2.3 Data analysis

All data were processed and visualized using Excel 2022 and OriginPro 2022. Each result is presented as the mean ± standard deviation (SD) of three parallel measurements. SPSS 26.0 software was used to perform one-way and two-way analysis of variance (ANOVA) to assess significant differences between different treatments. The significance level was set at *p* < 0.05. XRD data were analyzed using HighScore Plus software, and mineral composition images were created using OriginPro 2022 software.

This study used the above methods to investigate the effects of the indigenous fungi (J-Z) inoculant on nutrient transformation, mineral evolution, and organic acid production mechanisms in coal gangue and coal gangue-loess composite systems.

## 3 Results

### 3.1 Variations in J-Z fungi abundance

**Figure 2:**
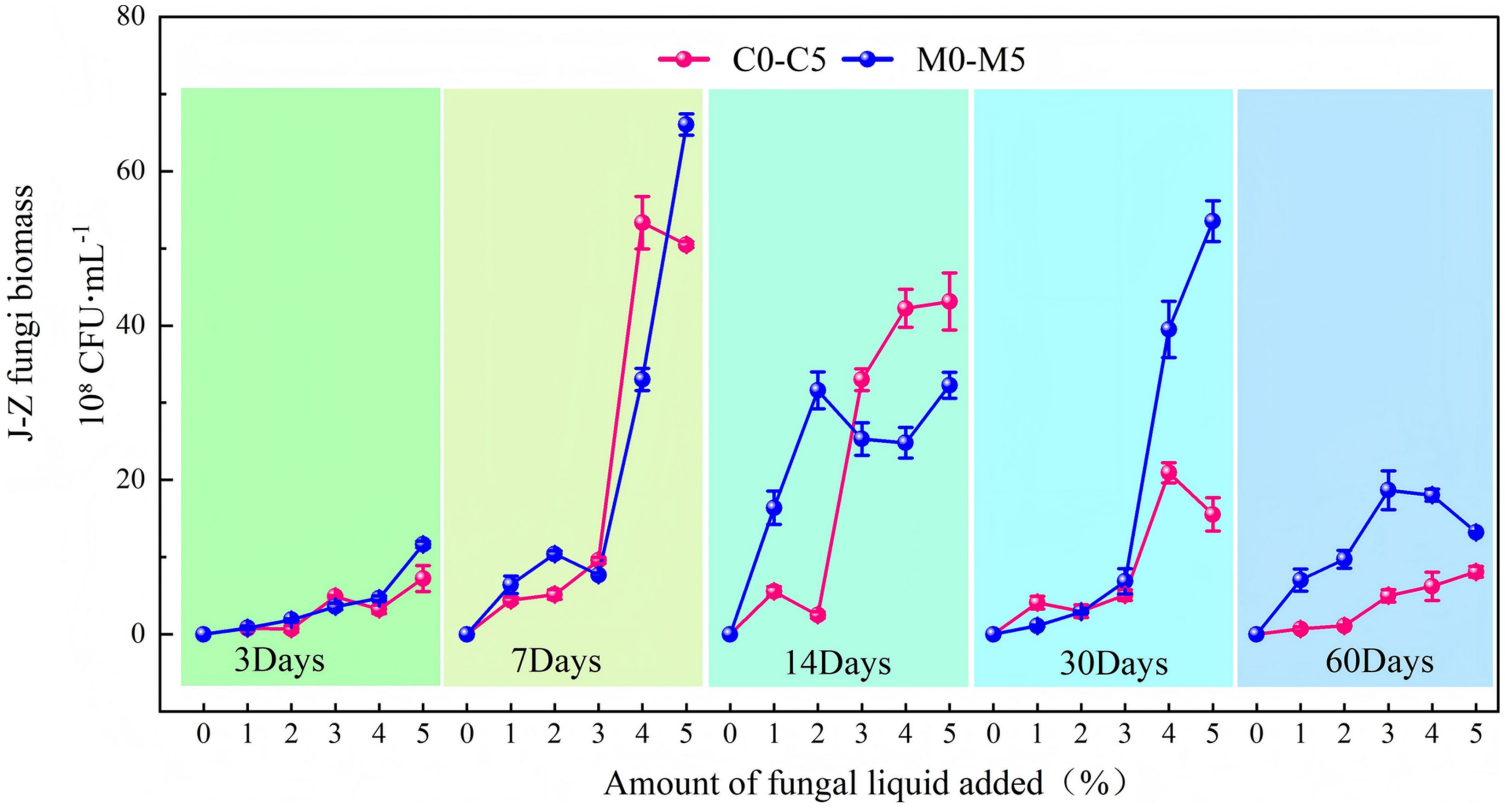
Changes in the number of indigenous fungi (J-Z) under different treatments. The C treatment consisted of pure coal gangue, whereas the M treatment represented the mixture of coal gangue and loess (80:20 g).

J-Z fungi abundance exhibited strong dependence on the inoculum concentration at each sampling stage (Figure 2). During the initial 3 days, both the C and M systems showed limited J-Z fungi growth at low inoculum levels.

A large increase was observed from day 3 to 7. J-Z fungi abundance increased with increasing inoculum levels, peaking with the 4–5% treatment (5.3 × 10^9^ CFU·mL^−1^ in C4 and 6.6 × 10^9^ CFU·mL^−1^ in M5). Therefore, both systems exhibited a short period of exponential growth. From day 14, the C system maintained relatively high J-Z fungi abundance (3.3–4.3 × 10 CFU·mL^−1^) with 3–5% inoculum level, while the M system peaked at moderate concentrations (2–4%). This indicates that loess may improve the physicochemical conditions of the substrate. On day 30, J-Z fungi abundance in M3 and M4 was significantly higher than that in C3 and C4, indicating that the matrix of the M system provided longer-term support for the J-Z fungi community. For the pure gangue system, the biomass peak at 4% was temporary. On day 60, the J-Z fungi populations in both systems decreased with the gradual consumption of nutrients. However, M3 and M4 still showed higher levels (up to 1.8 × 10^9^ CFU·mL^−1^) than C3 and C4, indicating that the environmental buffering ability of the mixed substrate was much better.

Comparing different incubation periods at the same inoculum level, J-Z fungi abundance with low-concentration inocula (1–2%) increased slowly during the first 3–7 d but kept accumulating in the M system during the subsequent 14–30 d (Figure 2), indicating that the relatively small inoculum inputs were enough to maintain J-Z fungi activities when loess was added. At moderate concentrations (3–4%), J-Z fungi abundance was higher and more stable in the M treatments during the subsequent 14–30 d, which could provide a more balanced and sustainable growth environment. At a high concentration (5%), J-Z fungi abundance in the C treatments peaked between the latter 7 and 14 d and then sharply decreased afterwards (30–60 days). Therefore, the substrate provided limited support for long-term survival. Although the J-Z fungi abundance in the M treatments also responded positively to the 5% inoculum in the early stage, the fluctuations in biomass were more pronounced in the later stage. Under excessive inoculum concentrations, the J-Z fungi community structure may be disturbed instead of stabilized.

Generally, the J-Z fungi abundance in the M treatment was always higher than that in the C treatment at most concentrations and sampling times. Loess may improve the overall substrate structure and buffering capacity, which is beneficial for the proliferation and long-term survival of J-Z fungi. Although high inoculum concentrations induced short-term stimulation in the C treatments, the biomass sharply decreased in the later stage (30–60 d).

### 3.2 Changes in pH and EC

**Figure 3:**
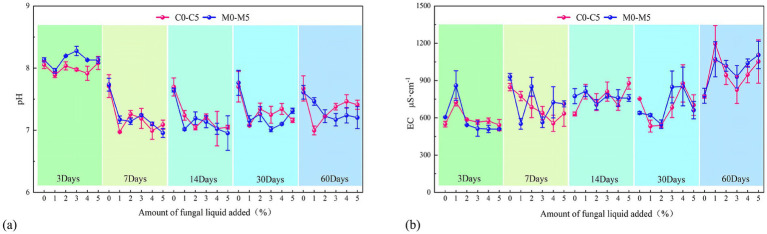
pH changes after adding indigenous fungi solution under different treatments **(a)**, Changes in EC after adding indigenous fungi solution under different treatments **(b)**, the C treatment consisted of pure coal gangue, whereas the M treatment represented the mixture of coal gangue and loess (80:20 g).

At the same sampling time but under different inoculum concentrations, the pH of both the C and M treatments remained around 8.0 on day 3, showing little variation with increasing inoculum concentration (Figure 3a), indicating that the J-Z fungi inoculum had little effect on pH during the early stage. By day 7, the pH of both systems decreased significantly to 7.0–7.5, reflecting an acidification process; the extent of change was relatively small among different concentrations. At day 14, pH stabilized at approximately 7.0, with minimal differences between treatments. A slight rebound of pH was observed on day 30 in some treatments (Figure 3a). For the M treatments with 3–4% inoculum, the pH was still relatively low and stable (7.0–7.3). For the other treatments, the pH was stable at 7.0–7.5 on day 60. The effect of inoculum concentration was no longer significant after day 60.

At various incubation periods with the same inoculum concentration, the pH of the C and M treatments, still alkaline on day 3, decreased rapidly between days 7 and 14, and then stabilized in the slightly alkaline or near-neutral range. For the two systems with 0–1% inocula, both showed this trend at first and then stabilized in the slightly alkaline or near-neutral range, reflecting the weak buffering effects during early inoculation. With 2–3% inocula, the pH of the M treatments was higher than that of the corresponding C treatments by day 30, indicating that the loess improved the buffering capacity of the two systems. With 4–5% inocula, the pH decreased sharply during early inoculation but then stabilized after day 14. No significant improvement was observed over inoculation with a moderate amount of loess.

The pH gradually decreased in the alkaline state and then stabilized at 7.0–7.3 during days 7–14. Compared to the C system, the M system could mitigate acidification to a certain degree, resulting in a higher and more stable pH at days 7–30. Among all treatments, the M3–M4 groups maintained the pH at 7.0–7.3 by day 30, representing the most favorable condition for maintaining a neutral buffered solution.

On day 3, the EC of both groups was 500–900 μs·cm^−1^, with only small variations for low-concentration treatments (0–2%, Figure 3b). However, EC increased greatly at higher inoculum levels, peaking in M5. On day 7, the differences among the C and M systems became more apparent. The 4–5% inocula generated the highest EC, suggesting that higher J-Z fungi activity promoted the dissolution and accumulation of soluble ions. With an incubation time of 14 days, the differences between the treatments gradually decreased, and the EC converged at 600–800 μs·cm^−1^, indicating that the rate of soluble salt release gradually decreased after the first stage of rapid mineral dissolution (day 7). With an incubation time of 30 days, the EC of medium- and high-concentration treatments (3–5%) increased again, especially for M4 and M5. With an incubation time of 60 days, the M3–M5 and C3–C5 groups peaked (EC: 1000–1,300 μs·cm^−1^) at day 60 and were much higher than those in the 0–2%-inoculum groups.

At the same inoculum concentration but varying incubation times, we observed limited fluctuations in EC with low-concentration inoculums (0–2%, EC: 500–800 μs·cm^−1^, throughout incubation). In medium-to-high-concentration treatments (3–5%), the EC showed a two-stage accumulation: EC increased rapidly on days 3–7, plateaued near day 14, and increased significantly from day 30.

The EC of the M treatments was always higher than that of the C treatments, and the differences became more distinct under medium- and high-concentration treatments and subsequent incubation stages (30–60 days), suggesting that loess addition improved the physicochemical conditions for J-Z fungi activity and enhanced the release of ions from the substrate. Among all the treatments, M4 showed optimal performance on days 30–60, with the highest EC sustained over the longest period. This indicates that the mixed substrate with 4% inoculum enhanced the dissolution of salt and the capacity for matrix stabilization.

### 3.3 Changes in total organic carbon (TOC) content

**Figure 4:**
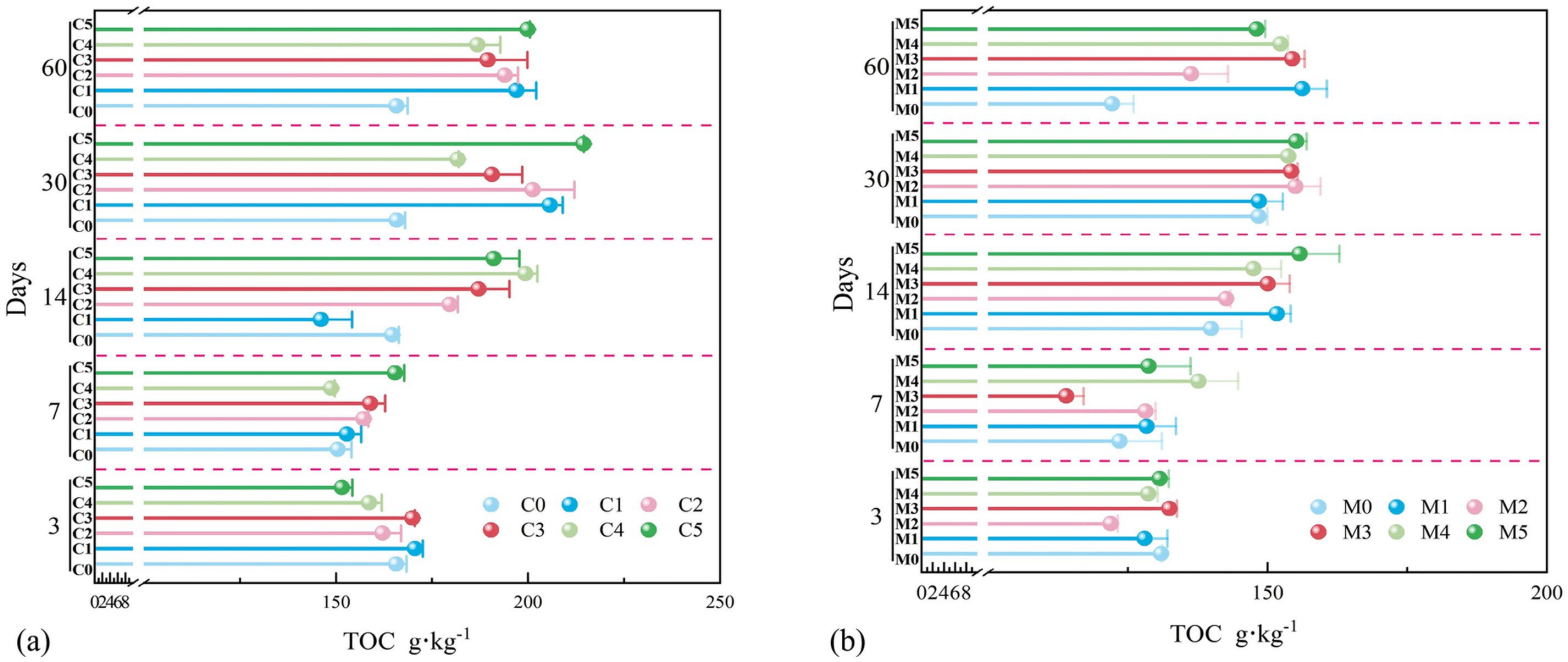
Changes in TOC after adding indigenous fungi solution, representing the C treatment of pure coal gangue **(a)**, and the M treatment of coal gangue mixed with yellow loam **(b)**.

C3–C5 had a marginally higher TOC content than C0 on day 3, whereas TOC varied only slightly among the C treatments on day 7 (Figure 4a), suggesting that a higher inoculum input may stimulate the initial accumulation of organic carbon. Indeed, TOC increased markedly with respect to C1–C2 and the control at day 7 (medium- to high-level inocula increased TOC enrichment in the early stages). On day 14, C3–C5 still showed clear advantages, especially C4 and C5, where TOC exceeded 200 g·kg^−1^. On day 30, TOC increased further for all fungal-inoculated treatments, and the difference in relation to the control was even greater owing to increased J-Z fungi activity and community stimulation. Finally, after 60 days, C5 presented the greatest TOC (>220 g·kg^−1^), followed by C3 and C4; thus, high inoculum concentrations increased TOC accumulation in the long term.

In terms of the incubation period, the TOC in C0 remained nearly unchanged throughout, demonstrating a limited capacity for organic carbon gain without inoculation. In contrast, C1–C2 showed minor improvements, whereas C3–C4 displayed pronounced increases between days 14 and 30 and remained elevated thereafter. C5 continued to accumulate TOC until day 60 and exhibited the strongest and most sustained enhancement. Overall, TOC content was positively associated with inoculum concentration, and medium- to high-level inocula (3–5%) proved most effective, particularly during 30–60 days. These results suggest that intensified J-Z fungi metabolism and organic matter formation contribute to improved carbon sequestration in coal gangue under high inoculum inputs.

In the M system, inoculation with the indigenous J-Z fungus generated a similar TOC pattern as in the C system (Figure 4b). However, owing to the dilution effect of the loess, the overall TOC content was lower. At the initial stage (3 days), TOC was ~130 g·kg^−1^, but gradually increased over time, showing more pronounced growth at 7 and 14 days, particularly for M5, where TOC rapidly increased to ~150 g·kg^−1^ before stabilizing. By days 30 and 60, TOC continued to increase slightly and stabilized around 160 g·kg^−1^. Under high concentrations, TOC increased in both systems [M3: 132 → 154 g·kg^−1^ (+16.67%), M4: 128 → 152 g·kg^−1^ (+18.75%), M5: 130 → 148 g·kg^−1^ (+13.85%); C3: 169 → 189 g·kg^−1^ (+11.83%), C4: 158 → 186 g·kg^−1^ (+17.77%), C5: 151 → 199 g·kg^−1^ (+31.79%)]. Although the TOC levels were lower in the M than in the C treatments, their increase was more consistent with reduced variability. At moderate inoculum concentrations (M3 and M4), TOC maintained relatively high growth rates, suggesting improved system stability.

In the C and M systems, inoculation with indigenous fungi significantly increased TOC accumulation. Higher concentrations of inocula can sustain the long-term enrichment of TOC. Although the addition of loess slightly reduced the TOC content, it enhanced the buffering capacity and stabilized the carbon accumulation process. Among all treatment groups, M4 achieved the optimal balance between long-term TOC improvement and physical and chemical stability, representing the optimal substrate configuration.

### 3.4 Changes in available phosphorus (AP) content

**Figure 5:**
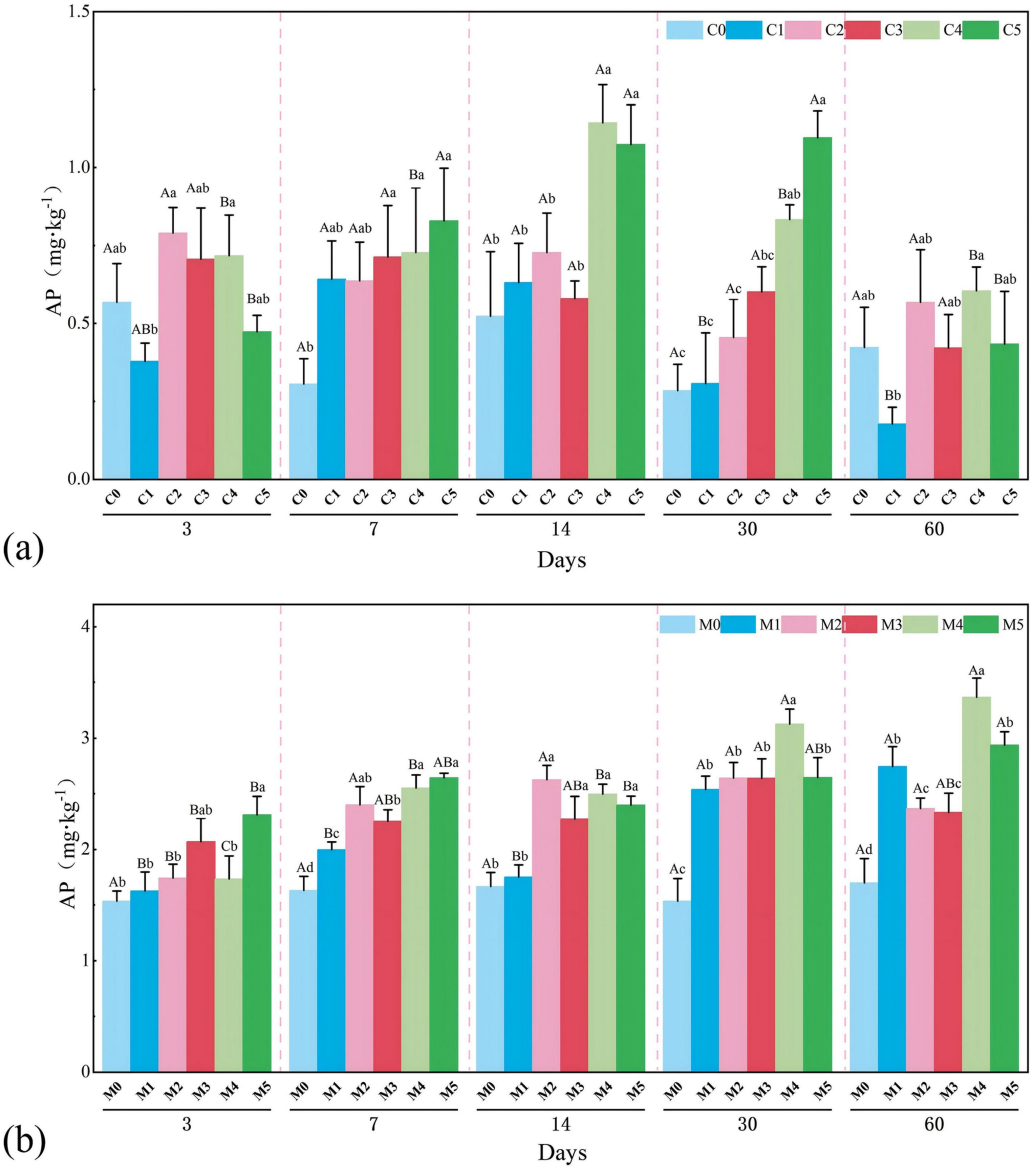
Changes in AP after adding indigenous fungi solution, represents the C treatment of pure coal gangue **(a)**, describes the M treatment of coal gangue mixed with yellow loam **(b)**, different lowercase letters indicate significant differences between treatments on the same day (*p* < 0.05). Different capital letters indicate significant differences between the same treatment over different days (*p* < 0.05).

In the C system, AP content was <1.0 mg·kg^−1^ on day 3, and C2–C4 had higher AP contents than C0, C1, and C5, indicating that a moderate inoculum concentration could stimulate early P release (Figure 5a). At day 7, AP content in C2–C5 was higher than that in the control, especially in C5, indicating that increasing the inoculum concentration enhanced phosphate solubilization (Figure 5a). AP content peaked on day 14, and C4 and C5 reached 1.0 mg·kg^−1^. AP content decreased after day 14; C4 and C5 still presented relatively high AP contents at day 30 (Figure 5a). On day 60, AP content decreased in all treatments (Figure 5a). However, AP contents in C2 and C4 were still higher than those in C0 (Figure 5a). In general, C4 exhibited the most stable performance, and its AP content was continuously enhanced and then gradually stabilized (Figure 5a).

In the M system, AP content was higher than that in the C system at any incubation time (Figure 5b). At day 3, AP content was 1.5–2.5 mg·kg^−1^ and increased with inoculum concentration; M4 and M5 had the highest AP content (Figure 5b). On day 7, AP content reached 2–3 mg·kg^−1^, and all treatments presented a clear concentration-dependent trend (Figure 5b). AP content peaked on day 14 and remained high until days 30–60 (Figure 5b). AP content in M1–M2 increased only slightly with time, and M4 presented the highest and most stable AP content until day 60, while M5 increased to 3.0–3.5 mg·kg^−1^ (Figure 5b).

Inoculation with indigenous fungi enhanced P release from coal gangue, and loess incorporation further improved P solubilization and retention. Medium-to-high concentrations (4–5%) were the most effective, and M4 (4%) presented the optimal balance between rapid improvement and long-term stability.

### 3.5 Mineral composition analysis

Among the treatments, M4 showed the greatest overall performance after 30 days. J-Z fungi abundance increased significantly from 4.67 × 10^8^ CFU·mL^−1^ to 2.48 × 10^9^ CFU·mL^−1^ during days 3–30, with high proliferation capacity and sustained community activity. The pH decreased significantly from 8.17 to 7.10 during days 3–30, and the EC was high and stable for days 30–60, indicating that mineral dissolution and ion release were sustained by the buffering effects of the substrate’s physicochemical properties. In addition, the nutrient properties showed clear improvement: TOC increased from 128 g·kg^−1^ to 152 g·kg^−1^ and AP from 1.73 mg·kg^−1^ to 3.37 mg·kg^−1^ during days 3–30. M4 achieved the optimal balance of J-Z fungi activation, physicochemical stabilization, and nutrient accumulation, making it the most suitable inoculum concentration for improving the reclamation potential of coal gangue–loess substrates.

**Figure 6:**
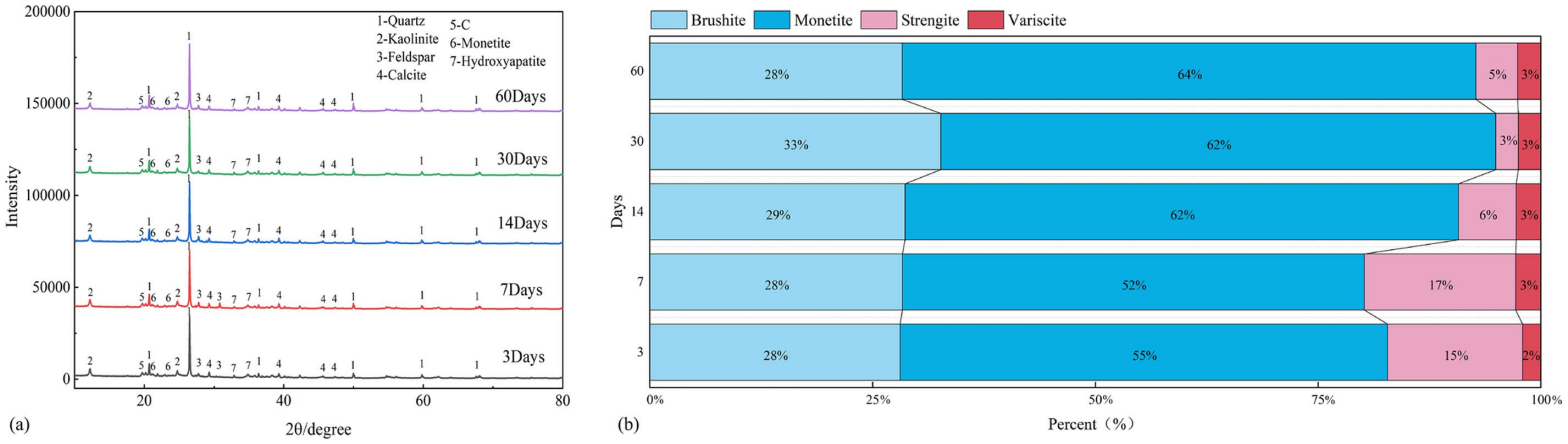
XRD spectra at different time intervals under M4 treatment **(a)**, Normalized comparison chart of phosphorus-containing minerals **(b)**.

To understand the mechanism responsible for the significant improvement in AP, we characterized the minerals in the M4 system. Figure 6a shows the XRD patterns of the M substrate inoculated with 4% inoculum at different periods (3, 7, 14, 30, and 60 d) and the corresponding variations in mineral composition. According to diffraction peak matching, the predominant P-containing minerals were brushite, monetite, strengite, and variscite. The phosphorus release potential of these minerals differed significantly, generally in the following order: brushite (high availability) > monetite (moderate availability) > strengite ≈ variscite (very low availability) (Adnan et al., 2025).

Brushite (CaHPO₄·2H₂O, dicalcium phosphate dihydrate), the most soluble phase, dissolves under neutral to mildly acidic conditions, releasing phosphate ions readily assimilated by plants and microorganisms (Giocondi et al., 2010; Coker et al., 2025). Therefore, brushite is a readily available source of P. The calcium phosphate mineral monetite (CaHPO₄, dicalcium phosphate anhydrous) is slightly less soluble than brushite and can gradually release available phosphorus, albeit at a slower rate, but remains much more bioavailable than iron/aluminum phosphates (Jaynes et al., 1999). Strengite (FePO₄·2H₂O, iron phosphate dihydrate) exhibits extremely low solubility due to the strong binding between Fe^3+^ and PO₄^3−^ (Rathnayake and Schwab, 2022). It typically acts as a phosphorus-fixing phase and is nearly unavailable to plants. Variscite (AlPO₄·2H₂O, aluminum phosphate dihydrate) has a solubility similar to that of strengite, with Al^3+^ strongly bound to phosphate ions (Saentho et al., 2022). As a typical “phosphorus-fixing” mineral, variscite also shows poor bioavailability.

Brushite and variscite showed minimal fluctuations throughout the incubation period, with only a slight increase in brushite observed at 30 days (Figure 6b). In contrast, monetite and strengite exhibited notable changes: monetite increased from 55% at 3 days to 64% at 60 days, whereas strengite decreased from 15% at 3 days to 5% at 60 days.

### 3.6 Analysis of organic acid metabolites

To further clarify the role of indigenous J-Z fungi in mineral phase transformation, the major organic acids present in the fungal inoculum—citric acid, L-pyroglutamic acid, fumaric acid, and malic acid—were analyzed to determine their potential contributions to phosphate dissolution (Zhu et al., 2023). As shown in Table 2, considering both their relative concentrations and capacities to induce mineral conversion, the four organic acids were ranked in the following order: citric acid > L-pyroglutamic acid > fumaric acid > malic acid (Xu et al., 2004).

**Table 2:** Test results of organic acids in the indigenous fungus.

Indicator	Content (ng·mL^−1^)	Indicator	Content (ng·mL^−1^)
Citric acid	2668.89 ± 202.534	Pantothenic acid	80.23 ± 3.987
Pyroglutamic acid	1506.58 ± 122.702	3-Methylglutaric acid	57.55 ± 2.528
Fumaric acid	1188.28 ± 63.124	Homovanillic acid	40.06 ± 5.655
Malic acid	876.2 ± 85.183	Phenylpyruvic acid	14.04 ± 2.99
5-Hydroxymethyl-2-furoic acid	319.81 ± 8.001	Adipic acid	12.48 ± 0.783
Malonic acid	291.62 ± 11.087	Phenyllactic acid	12.41 ± 0.688
3-Hydroxy-3-methylglutaric acid	239.92 ± 15.672	D-Glucuronic acid	12 ± 2.407
Lactic acid	132.28 ± 1.101	Nicotinic acid	7.68 ± 0.79
Glutaric acid	118.96 ± 8.802	Suberic acid	5.79 ± 0.301
Tartaric acid	106.67 ± 4.961	Ethylmalonic acid	1.65 ± 0.009

### 3.7 Phosphorus activation and mineral transformation mechanism

Citric acid has a tricarboxylic structure and high Fe^3+^ complexation capacity, which can compete with Fe^3+^ in strengite and break the Fe–P bond. From here, the phosphate ion reprecipitates with Ca^2+^ to form monetite (Sun et al., 2025). Citric acid can change the mineral equilibria of indigenous communities and enhance phosphorus release, likely through protonation and strong Fe^3+^ chelation. L-Pyroglutamic acid has only one carboxyl group; its Fe^3+^ complexation is weak, and the acidification effect is also very poor, which cannot promote the transformation from strengite to monetite (Sun et al., 2025). Fumaric acid has a rigid structure; its Fe^3+^ chelation ability is moderate, its acidification ability is also moderate, and its ability to induce changes in the mineral phase is limited (Gancel et al., 2022). Compared with the other two dicarboxylic acids, malic acid has one more hydroxyl group, and its Fe^3+^-binding ability is moderate, which can partially enhance phosphorus release, though at lower levels than citric acid (Ma et al., 2020).

Citric acid mobilizes phosphorus from strengite through combined protonation and strong Fe^3+^ chelation (Gracheva et al., 2024). The liberated phosphate can subsequently reprecipitate with Ca^2+^to form Monetite or its hydrated derivatives when calcium availability is sufficient.

Fe - P bonds weaken and strengite undergoes protonation and dissolution under acidic conditions (represented by H^+^ in the environment):


$$F\mathrm{eP}O_{4}\cdot 2H_{2}O(s)+2H^{+}⇌Fe^{3+}+\mathrm{HP}O_{4}^{2-}+2H_{2}O$$

Citric acid forms complexes with Fe^3+^ (capturing and dissolving Fe^3+^, stabilizing iron, and maintaining phosphorus in solution):


$$Fe^{3+}(\mathrm{aq})+\mathrm{Ci}t^{3-}(\mathrm{aq})⇌\mathrm{Fe}(\mathrm{Cit})(\mathrm{aq})$$

Phosphate ions in the solution combine with Ca^2+^ to form monetite (precipitate):


$${\mathrm{Ca}}^{2+}(\mathrm{aq})+{\mathrm{HPO}}_{4}^{2-}(\mathrm{aq})\to {\text{CaHPO}}_{4}(s)(\text{Monetite})$$

Overall, the process can be described as follows: citric acid (Cit^3−^) complexes with and dissolves Fe^3+^, while the released HPO₄^2−^ combines with Ca^2+^ in solution to precipitate as Monetite (CaHPO₄).

Through systematic optimization of J-Z fungi inoculum concentration, this study established a coupled mechanism framework of “J-Z fungi activation - mineral transformation - nutrient stabilization” within the coal gangue - loess system. The findings not only deepen the understanding of J-Z fungal-driven mineral evolution and nutrient activation processes but also provide a practical technical pathway for the resource utilization of coal gangue and ecological reclamation of mining areas.

## 4 Discussion

### 4.1 Influence of J-Z fungi additive dosage on J-Z fungi dynamics

The C system with high inoculum concentration (4–5%) exhibited rapid J-Z fungi proliferation during the subsequent 7–14 days and then sharply decreased afterwards, likely due to the rapid depletion of substrate nutrients and intense J-Z fungi competition. Similarly, Wang et al. (2024c) reported that the inoculation of *Bacillus velezensis* in coal-based media promoted the solubilization of nutrients, though the beneficial effects disappeared beyond 5% and, consequently, nutrient release decreased.

In contrast, the M system sustained high J-Z fungi abundance for a longer duration, particularly at moderate inoculum concentrations (3–4%). The addition of loess likely enhanced the physicochemical environment by improving the buffering capacity and supplying trace nutrients, collectively establishing a more favorable habitat for J-Z fungi proliferation and persistence. Similarly, Wang et al. (2024b) study confirmed that the introduction of loess enhanced J-Z fungi colonization and delayed biomass decline. Therefore, the combination of a moderate inoculum level with a loess-based matrix can optimize system bioactivity and stability.

We found that a moderate inoculum concentration (4%) not only enhanced J-Z fungi activity but also stabilized the system’s physicochemical characteristics and promoted nutrient cycling. Compared to previous findings of Shi et al. (2025), where “high-dose treatments stimulated short-term growth but led to a later-stage decline,” the M4 treatment exhibited better long-term stability with sustained effects.

### 4.2 Effects on pH and EC: buffering and ion exchange mechanisms

The pH showed slight acidification between days 7 and 14 in treatments inoculated with fungi and then stabilized. The stabilization was highly evident in the M3 and M4 treatments. The overall trend was probably due to the combined effects of (1) organic acids released by microbes and (2) carbonate in buffering the water solution. The dissolution of CaCO₃ and the cation exchange process in the loess component may retard acidification in the culture solution. Similarly, Yang et al. (2022) showed that substrates rich in carbonate can alleviate the acidification in the culture solution caused by the metabolism of microorganisms.

The increase in EC at higher inoculum concentrations indicated that J-Z fungi metabolism could enhance mineral dissolution and release more ions into the solution, which may be stimulated by the J-Z fungi production of organic acids and chelating compounds. The relatively stable EC for M4 indicated that the mixed system maintained a balance between dissolution and reprecipitation, thereby sustaining ion release without ion accumulation. Newsome and Falagán (2021) have also shown that appropriate J-Z fungi inoculation helps maintain ion balance, preventing excessive ion release that could lead to salt accumulation or nutrient loss.

### 4.3 Organic carbon accumulation and J-Z fungi-driven organic matter transformation

The results showed that inoculation with the indigenous fungal strain J-Z significantly increased TOC accumulation, and the effects in the M3 and M4 treatment groups were the most significant. Therefore, the inoculum helps stabilize organic carbon and convert soluble carbon components into more stable organic matter. Similarly, Barapatre et al. (2021) found that J-Z fungi inoculation in a coal gangue-loess system promoted the formation of humus-like substances and enhanced the combination of carbon minerals. Therefore, the optimal inoculation amount appears to balance J-Z fungi metabolism and organic matter turnover, whereas excessive inoculation may promote carbon loss.

### 4.4 Phosphorus activation and mineral transformation mechanism

The results showed that inoculation with indigenous J-Z fungi successfully transformed the insoluble iron ore into a more easily weathered substrate. This transformation in mineral composition is highly consistent with the significant increase in phosphorus adsorption, indicating that J-Z fungi gradually enhance the migration ability of phosphorus by converting insoluble iron phosphate into soluble calcium phosphate.

In the M system, AP content was significantly increased by M4 treatment (3.0–3.5 mg·kg^−1^ at 60 d). XRD revealed that the poorly soluble iron phosphate strengite was progressively converted to monetite by J-Z fungi activity, representing a J-Z fungi-mediated phosphorus activation process that improved long-term P availability.

The improved phosphorus availability could be mainly attributed to the J-Z fungi’s secretion of organic acids, such as citric acid. Its tricarboxylic structure exhibits a strong chelating ability that can break the Fe^3+^–P bond and release PO_4_^3−^, which reacts with Ca^2+^ to form monetite. Menezes-Blackburn et al. (2016) and Wang et al. (2022) reported a similar dissolution–reprecipitation process, reflecting the function of citric acid as an efficient ligand for Fe–P mineral dissolution. Moreover, Hu and Chen (2023) found that phosphate-solubilizing fungi can mobilize phosphorus while reducing heavy metal mobility by secreting organic acids. Consistent with these studies, the present work showed similar mechanisms of strengite dissolution and monetite formation, as evidenced by the direct XRD results.

Changes from the Fe–P to Ca–P phases suggest that indigenous communities can change mineral equilibria to more available phases to promote resource use. Similarly, Silva et al. (2023) proposed that phosphate-solubilizing microorganisms are more prominent because they maintain the phosphorus cycle; therefore, their presence must ideally be maintained to optimize ecological restoration and nutrient enrichment.

## 5 Conclusion

In this study, the effects of different concentrations of indigenous fungi (J-Z) agents on the physicochemical properties and nutrient activation of coal gangue and its mixed system with loess were systematically evaluated. The results showed that the treatment of medium concentration of fungi solution was the best, which could significantly promote J-Z fungi reproduction, reduce pH, increase EC, and tend to be stable, and increase TOC and AP content. The organic acids secreted by indigenous fungi (J-Z) promoted the transformation of the difficult-to-use phosphorus-containing mineral strengite to the easy-to-use phosphorus-containing mineral monetite, and achieved the activation of available phosphorus. In general, the addition of 4% J-Z indigenous fungus liquid after the mixing of coal gangue and raw loess has both efficient nutrient release and stability, which is the best treatment for the resource utilization of coal gangue and soil-like materials.

The comprehensive performance evaluation indicated that the 4% inoculation treatment achieved an effective balance between nutrient activation and system stability. This approach not only enhanced substrate fertility but also maintained stable physicochemical conditions, making it suitable for improving coal gangue–based substrates. Under controlled conditions, this treatment is therefore recommended for environmental restoration applications involving materials with similar characteristics.

## Data Availability

The original contributions presented in the study are included in the article/supplementary material, further inquiries can be directed to the corresponding author.
